# MTERF3 contributes to MPP+-induced mitochondrial dysfunction in SH-SY5Y cells: MTERF3 in mitochondrial dysfunction

**DOI:** 10.3724/abbs.2022098

**Published:** 2022-07-28

**Authors:** Shun Zhu, Nan Xu, Yanyan Han, Xiaofei Ye, Ling Yang, Ji Zuo, Wen Liu

**Affiliations:** Department of Cellular and Genetic Medicine School of Basic Medical Sciences Fudan University Shanghai 200032 China

**Keywords:** MTERF3, mitochondrial dysfunction, degenerative disease, environmental toxins, Parkinson’s disease

## Abstract

Parkinson’s disease (PD) is a neurodegenerative disorder causing severe social and economic burdens. The origin of PD has been usually attributed to mitochondrial dysfunction. To this end, mitochondrial transcription regulators become attractive subjects for understanding PD pathogenesis. Previously, we found that the expression of mitochondrial transcription termination factor 3 (MTERF3) was reduced in MPP+-induced mice model of PD. In the present study, we probe the function of MTERF3 and its role in MPP+-induced cellular model of PD. Initially, we observe that MTERF3 expression is also reduced in MPP+-induced cellular model of PD, which can be mainly attributed to the increase of MTERF3 degradation. Next, we examine the effect of MTERF3 knockdown and overexpression on the replication, transcription, and translation of mitochondrial DNA (mtDNA). We show that knockdown and overexpression of MTERF3 have opposite effects on mtDNA transcript level but similar effects on mtDNA expression level, in line with MTERF3’s dual roles in mtDNA transcription and translation. In addition, we examine the effect of MTERF3 knockdown and overexpression on mitochondrial function with and without MPP+ treatment, and find that MTERF3 seems to play a generally protective role in MPP+-induced mitochondrial dysfunction. Together, this work suggests a regulatory role of MTERF3 in MPP+-induced cellular model of PD and may provide clues in designing novel therapeutics against PD.

## Introduction

Parkinson’s disease (PD) is a common neurodegenerative disorder characterized by progressional loss of dopamine neurons in substantia nigra [1]. Researchers have shown that PD is resulted from a combination of genetic factors and environmental factors such as exposure to toxins like MPTP and paraquat [ 2– 4] . Although the etiology of PD is not completely understood, the role of mitochondrial dysfunction in PD pathogenesis has attracted increasing attention in recent years [ 5– 8] . Of the many regulators of mitochondrial biogenesis and homeostasis, the mitochondrial transcription termination factor (MTERF) family is of particular importance in that MTERF proteins function in the transcriptional regulation of mtDNA [ 9, 10] .

MTERF3 is an important member of the MTERF family. It has been shown that MTERF3 functions as a negative regulator of mtDNA transcription, as tissue-specific knockout of *MTERF3* in mice heart boosts the transcription of mtDNA significantly [11]. MTERF3 also functions as a regulator of mitochondrial ribosome biogenesis, as tissue-specific knockout of *MTERF3* in mice heart inhibits the assembly of 39S mitochondrial ribosome [12]. Furthermore, MTERF3 expression has also been found to be negatively correlated with the PD-related *PINK1* gene [13].

Despite the revealed function of MTERF3, a complete elucidation of its role in the disease state is far from complete. In this study, we probed the potential role of MTERF3 in mitochondrial dysfunction. SH-SY5Y cells were treated with mitochondria-targeting environmental toxins, 1-methyl-4-phenylpyridinium ion (MPP+) to construct the cellular model of PD. MPP+ has been shown to inhibit the activity of mitochondrial complex I, leading to insufficient ATP synthesis, loss of mitochondrial membrane polarity, and severe mitochondrial damages [ 14– 20] . Initial experiments by our group showed that while causing mitochondrial dysfunction, MPP+ also induces downregulation of MTERF3 expression in SH-SY5Y cells. By introducing a series of cellular inhibitors, we found that the MPP+-induced MTERF3 downregulation in SH-SY5Y cells is likely due to the increase of MTERF3 degradation. Furthermore, knockdown and overexpression of MTERF3 in MPP+-treated cells showed that MTERF3 seems to play a protective role in MPP+-induced mitochondrial dysfunction. This study reveals a potential role of MTERF3 in MPP+-induced cellular model of PD, laying the foundation for the development of novel therapeutics for PD.

## Materials and Methods

### Plasmid constructions

The full-length human *MTERF3* cDNA was kindly provided by Prof Jiahuai Han (Xiamen University, Xiamen, China). MTERF3 gene was cloned into the pLVX-IRES-Puro vector (Clontech, Mountain View, USA) for overexpression experiments using the following primers: MTERF3 forward 5′-ACGGGATCCGCCACCATGGCTTTGTCAGCCCAAC-3′ and reverse 5′-CCGCTCGAGCTAAAGCGTTTTTAAGAATTTTTCAAAG-3′.

For MTERF3-specific knockdown experiments, recombinant lentivirus-mediated shRNA interference was used. The RNA interference vector was made by inserting shRNA sequence into the pLVX-shRNA1 vector (Clontech). An shRNA was designed to target the MTERF3 sequence: 5′-TCAAGTTCCCACAGGTATTTA-3′. This RNAi sequence was obtained from the RNAi consortium shRNA library ( https://portals.broadinstitute.org/gpp/public/gene/search).The shRNA oligonucleotides were annealed and cloned into the pLVX-shRNA1 vector containing a puromycin resistance cassette and a *Bam*HI/ *Eco*RI fragment according to the manufacturer’s protocol, followed by sequence verification. A scramble shRNA targeting the sequence 5′-CCTAAGGTTAAGTCGCCCTCG-3′ was used as a negative control [21].

### Cell culture and transfection

Both HEK293T cells (Shisheng, Shanghai, China) and SH-SY5Y cells (kindly provided by Prof Fang Huang from Fudan University, Shanghai, China) were cultured in Dulbecco’s modified Eagle’s medium (DMEM; Gibco, Carlsbad, USA) supplemented with 10% fetal bovine serum (BioWest, Nuaillé, France) in an incubator at 37°C with 5% CO _2_. The sequence-verified MTERF3 overexpression or RNA interference plasmids with the lentivirus packaging plasmids psPAX2 or pMD2.G were cotransfected into HET293T cells using the transfection reagent Hilymax (Dojindo, Tokyo, Japan) following the manufacturer’s protocol. The lentiviral supernatants were harvested at 48 h after transfection and subsequently filtered using a 0.45 μm filter to obtain purified lentiviral solution, which was used to infect SH-SY5Y cells. SH-SY5Y cells were collected and screened at 72 h after infection using puromycin (Sigma, St Louis, USA) selection.

### Cell proliferation assay

To measure the cell viability, CCK-8 cell proliferation kit (Dojindo, Tokyo, Japan) was used. Cells were diluted to a concentration of 10 ^3^ cells per 100 μL, distributed to the 96-well plate (6 wells, 100 μL per well) and placed in an incubator at 37°C with 5% CO _2_ overnight. From day 1 to day 6, 10 μL of CCK-8 reagent was added into each well and placed in an incubator at 37°C with 5% CO _2_ for 1 h before measuring the absorbance at 450 nm.

### Cell cycle analysis

The proportion of cells within each stage of the cell cycle was determined using Cell Cycle kit (Beyotime, Shanghai, China) according to the manufacturer’s protocol. Briefly, the cells were harvested and fixed in 70% ethanol for 2 h at 4°C. The cells were then washed twice with cold PBS and treated with a mixture of RNase A and propidium iodide for 30 min at 37°C. Then, the proportion of cells at different stage of the cell cycle was analyzed on a BD Accuri™ C6 flow cytometer (BD Bioscience, Seattle, USA).

### Quantification of mtDNA and mRNA levels

Total DNA was extracted from the cells using a Wizard genomic DNA purification kit (Promega, Madison, USA). The relative mtDNA level was determined by quantitative real-time PCR, with the *TK2* gene as an internal standard and D-loop as a mtDNA marker that has been previously mentioned [ 20, 22] . Total RNA was extracted from the cells using a total RNA kit (TIANGEN, Beijing, China), and the cDNA was synthesized using a RevertAid first strand cDNA synthesis kit (Thermo Scientific, Waltham, USA). The relative mRNA level was determined using quantitative real-time PCR.

All quantitative real-time PCR experiments were performed on a MiniOpticon real-time PCR detection system (Bio-Rad, Hercules, USA) using AccuPower 2× Greenstar qPCR Master Mix (Bioneer, Daejeon, South Korea). The relative mtDNA and mRNA levels were calculated using the cycle threshold method [23]. Primers for mtDNA and mRNA quantifications are listed in Table 1.

**Table:** **Table 1** Primer sequences for mtDNA and mRNA quantifications

Gene	Forward (5′→3′)	Reverse (5′→3′)	Application
*TK2*	TCCTGCAGATGCCACTTTGA	CCCCAAGTCTGAAGAAAACG	mtDNA RT-PCR
*Dloop*	CATCTGGTTCCTACTTCAGGG	TGAGTGGTTAATAGGGTGATAGA	mtDNA RT-PCR
*MTERF3*	CAGTCTGCTTCCTTCCATGA	GCCTCTTCCTCTGAAATTGG	mRNA RT-PCR
*ND4L*	CGCTCACACCTCATATCCTC	CGGCAAAGACTAGTATGGCA	mRNA RT-PCR
*ND6*	CCCATCATACTCTTTCACCCA	GGGTTGAGGTCTTGGTGAGT	mRNA RT-PCR
*MTCO1*	CGATGCATACACCACATGAA	TCCAGGTTTATGGAGGGTTC	mRNA RT-PCR
*CYTB*	GCCTGCCTGATCCTCCAAAT	AAGGTAGCGGATGATTCAGCC	mRNA RT-PCR

### Western blot analysis

SDS-PAGE and western blotting were performed with standard protocols to detect protein expression levels using the following antibodies: anti-MTERF3 antibody (1:1000; Abcam, Cambridge, UK), anti-actin antibody (1:20,000; Sigma), anti-VDAC antibody (1:1000; Affinity, Cincinnati, USA), total OXPHOS rodent WB antibody cocktail (1:500; MitoSciences, Eugene, USA), anti-GAPDH antibody (1:10000; Sigma), and anti-β-actin antibody (1:5000; Sigma). Secondary antibodies included HRP-conjugated anti-mouse IgG (1:5000; Jackson, West Grove, USA) and HRP-conjugated anti-rabbit IgG (1:5000; Jackson). For experimental groups, cells were pre-treated with the following cellular inhibitors: zVAD (Beyotime, Shanghai, China), NH4Cl (Sinopharm, Shanghai, China), chloroquine (Sigma), MG132 (Sigma), and cycloheximide (Sigma).

### Immunofluorescence microscopy

Immunofluorescence experiments were performed with standard protocols using the following antibodies to label MTERF3 and COX IV: anti-MTERF3 (1:200; Abcam), and anti-COX IV (1:200; Abcam). Secondary antibodies used were Alexa Fluor 594-labeled donkey anti-rabbit IgG (1:300; Life Technologies, Carlsbad, USA) and Alexa Fluor 488-labeled rabbit anti-mouse IgG (1:2500; Life Technologies).

### Reactive oxygen species (ROS) and mitochondrial membrane potential assays

DCFH-DA probe experiments were performed to measure the ROS level in cells because reactive oxygen in cells oxidizes DCFH to produce fluorescent DCFs. DCFH-DA was diluted with serum-free culture medium at 1:1000 to a final concentration of 10 μM. SH-SY5Y cells were suspended in the diluted DCFH-DA solution at 10 ^6^–10 ^7^ cells/mL, and incubated in a incubator at 37°C for 20 min. The mixture was shaken every 3-5 min to fully mix the probe and the cells. Then the cells were washed three times with serum-free cell culture medium to remove free DCFH-DA. Then fluorescence intensity was measured with a Leica fluorescence microscope (Leica, Wetzlar, Germany) at a excitation wavelength of 488 nm.

JC-1 probe experiments were performed to measure the mitochondrial membrane potential. When the mitochondrial membrane potential is high, JC-1 forms polymers in the mitochondrial matrix, producing red fluorescence. When the mitochondrial membrane potential is low, JC-1 stays in monomers, producing green fluorescence. The transition from red fluorescence to green fluorescence indicates mitochondrial membrane depolarization. JC-1 dye solution was made by diluting the concentrated JC-1 (200×) and dispensing into 24-well plates with 250 μL per well. After the cells were washed with PBS buffer, JC-1 dye solution was added to the wells and incubated at 37°C for 20 min. After being washed with JC-1 dye buffer, cells were collected and observed under a Leica fluorescence microscope (Leica).

### ATP detection

To measure the cellular ATP level, the ATP assay kit (Beyotime) was used. The control group and experimental group of SH-SY5Y cells were treated with MPP+respectively at a final concentration of 2 mM. After 24 h, the lysis buffer was added to fully lyse the cells. After centrifugation at 4 °C and 12,000 *g* for 5 min, the supernatant was extracted for subsequent measurement. A total of 100 μL of ATP detection solution was added into each well of 96-well plates and the plate was left at room temperature for 5 min to remove background ATP. A total of 100 μL of sample solution or standard solution was then added into the detection wells. Next, the relative light unit (RLU) values were determined using a luminometer at least 2 s after the previous step. The ATP concentration was calculated using the standard curve.

### Statistical analysis

Data are presented as the mean±SEM. Statistical differences between groups were tested using an unpaired two-tailed Student’s *t*-test with GraphPad Prism software. *P*<0.05 was considered as statistically significant.

## Results

### MTERF3 is downregulated in MPP+-treated SH-SY5Y cells

**Figure 1:**
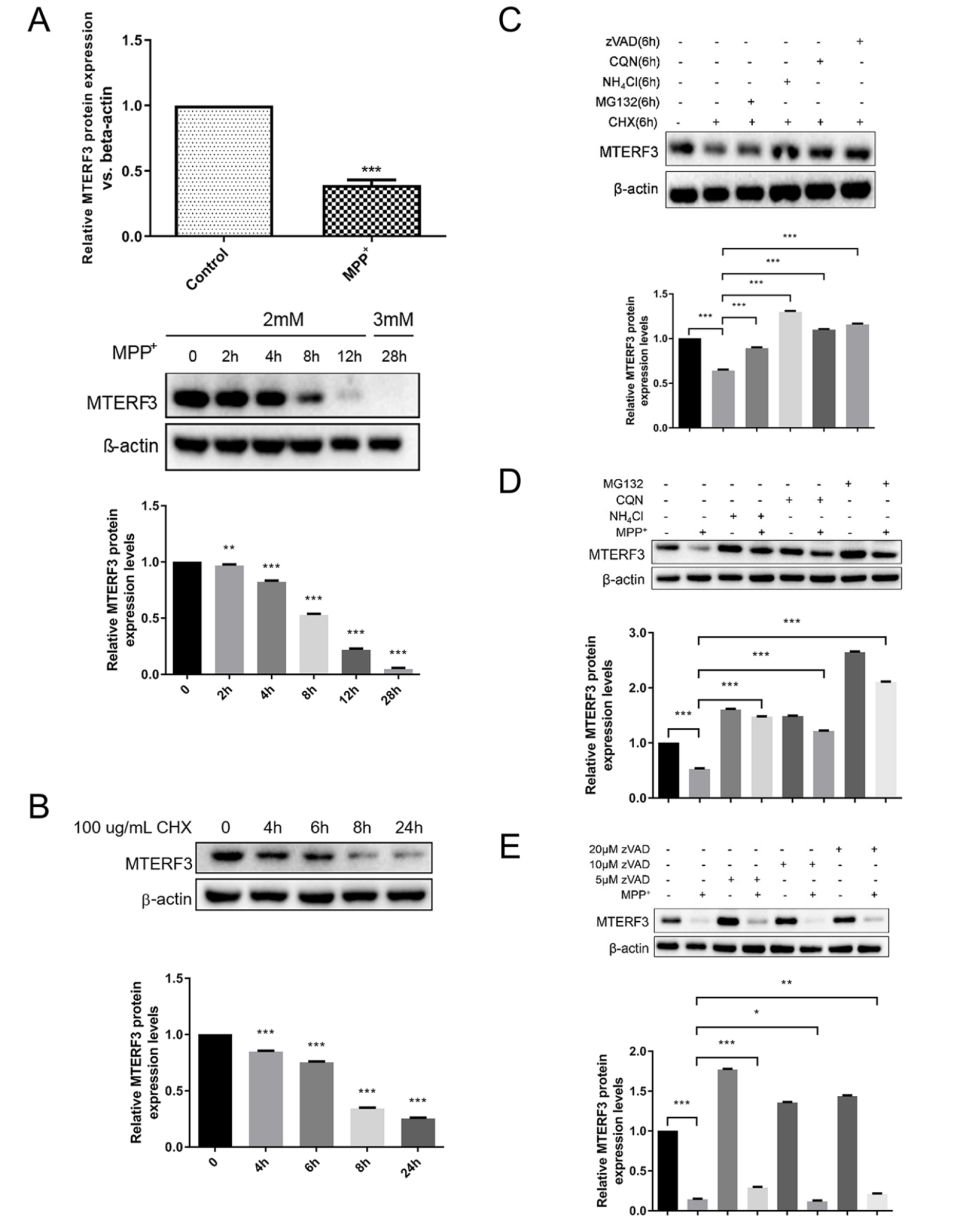
Figure 1 MTERF3 is downregulated upon MPP+ treatment in SH-SY5Y cells (A) Effects of MPP+ treatment on MTERF3 expression. (B–E) Effects of treatment with cellular inhibitors on MTERF3 expression. Data are presented as the mean±SEM, n=3. * P<0.05, ** P<0.01, *** P<0.001. ns: not significant.

Previously, we found that MTERF3 was significantly downregulated upon MPP+ treatment in the mouse model of PD [24]. In this study, to examine the role of MTERF3 in the cellular model of PD, we treated SH-SY5Y cells with the environmental toxin MPP+. Western blot analysis results indicated that 12 h of MPP+treatment caused a marked decrease of MTERF3 expression in SH-SY5Y cells ( Figure 1A, upper panel). A time-course experiment showed that the decrease of MTERF3 expression upon MPP+ treatment starts to accelerate at 4 h post treatment ( Figure 1A, middle and lower panels).

To find out the origin of MTERF3 downregulation, we examined MTERF3 expression after single or combined treatment with cellular inhibitors for apoptosis (zVAD), lysosome (chloroquine, *i*. *e*., CQN and NH4Cl), proteasome (MG132), and protein synthesis (cycloheximide, *i*. *e*., CHX). While CHX induced noticeable decreases in MTERF3 expression, zVAD, CQN, NH4Cl, and MG132 seemed to rescue the effect of CHX, suggesting that when protein synthesis is inhibited, MTERF3 enters a series of cellular degradation pathways, including the apoptotic pathway, the lysosomal pathway, and the ubiquitin-proteasome pathway ( Figure 1B,C). Next, we added MPP+ into the cells together with cellular inhibitors of various cellular degradation pathways. It appears that the MPP+-induced MTERF3 downregulation can be effectively rescued by the inhibitors of the lysosomal pathway or the ubiquitin-proteasome pathway but not by the inhibitor of the apoptotic pathway ( Figure 1D,E), suggesting that MPP+-induced reduction of MTERF3 expression is mainly due to the increase of protein degradation through the lysosomal and proteasome pathways.

### MTERF3 is located in the mitochondria

**Figure 2:**
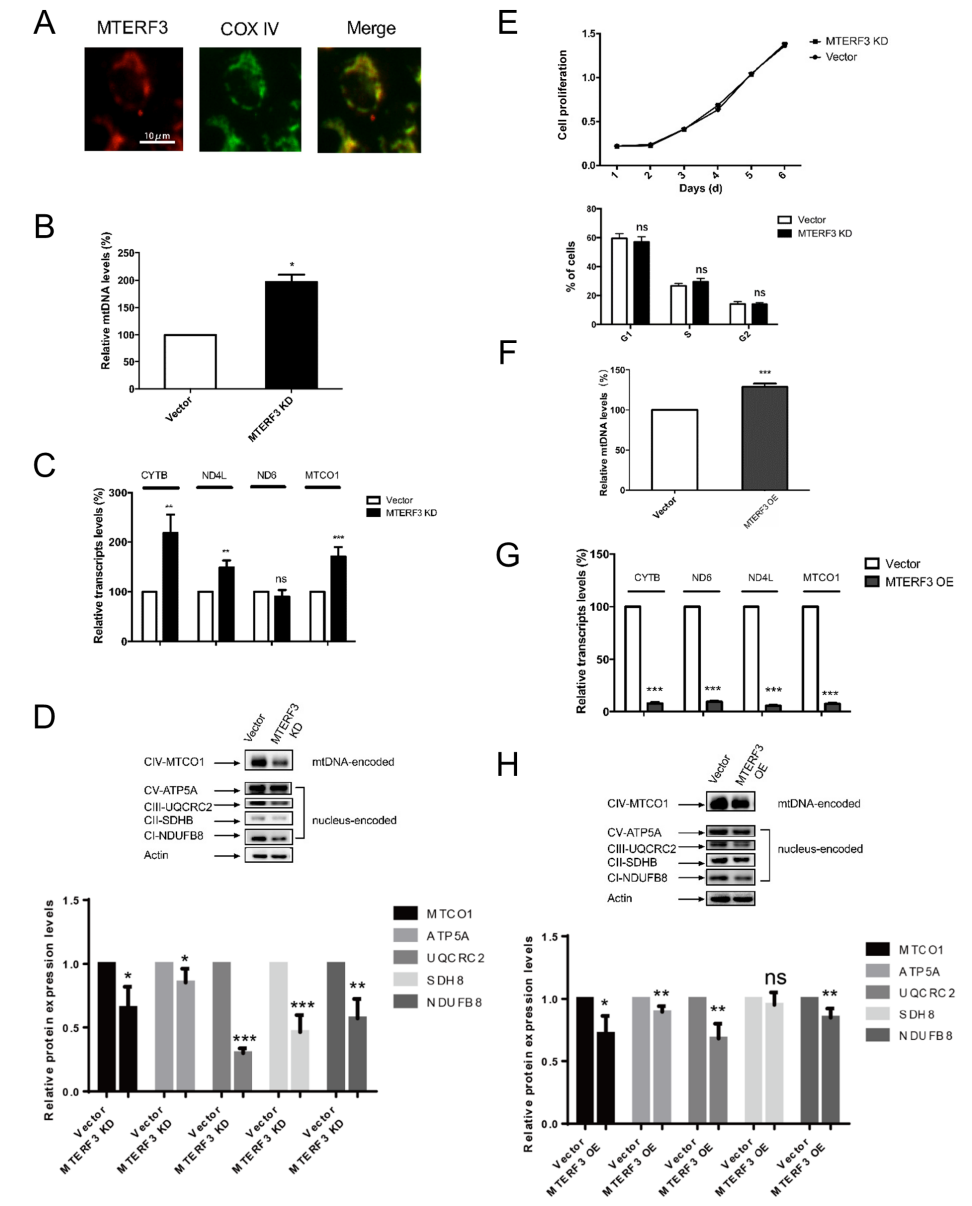
Figure 2 Regulatory effects of MTERF3 on mtDNA (A) MTERF3 colocalization with the mitochondrial marker COXIV. MTERF3 and COX IV are labeled in red and green fluorescence, respectively. (B–D) Effects of MTERF3 knockdown on mtDNA, mRNA, and protein levels. (E) Effects of MTERF3 knockdown on cell growth and cell cycle. (F–H) Effects of MTERF3 overexpression on mtDNA, mRNA, and protein levels. Data are presented as the mean±SEM, n=3 or 4. * P<0.05, ** P<0.01, *** P<0.001. ns: not significant.

The above experiments suggested that MTERF3 might be a potential player in MPP+-induced mitochondrial dysfunction. Subsequently, to confirm the subcellular location of MTERF3 in mitochondria, we monitored its colocalization with COX IV, which is a subunit of the nuclear DNA-encoded and mitochondria-located cytochrome c oxidase and an excellent marker for mitochondria. Superposition of the MTERF3 and COX IV fluorescence clearly showed that the red Alexa Fluor 594-labeled MTERF3 colocalized well with the green Alexa Fluor 488-labeled COX IV ( Figure 2A), suggesting that MTERF3 is a native resident protein of mitochondria, consistent with previous findings [11].

### MTERF3 regulates the replication, transcription, and translation of mtDNA

To elucidate the function of MTERF3, we initially performed knockdown (KD) experiments using MTERF3 shRNA in SH-SY5Y cells and monitored the replication, transcription, and translation of mtDNA. First, mtDNA level of the MTERF3-KD group was measured by quantitative real-time PCR. The mtDNA level showed a 2-fold increase after MTERF3 knockdown ( Figure 2B), which is probably due to the relief of MTERF3-mediated repression of mtDNA replication. Second, mRNA levels of four representative mtDNA-encoded mitochondrial proteins, *CYTB*, *ND4L*, *ND6*, and *MTCO1* were measured by quantitative real-time PCR. Compared with those of the control group, mRNA levels of *CYTB*, *ND4L*, and *MTCO1* in MTERF3-KD groups were increased by approximately 118%, 48.7%, and 71% respectively, consistent with the negative regulator role of MTERF3 in mtDNA transcription, although the change of *ND6* mRNA level was not apparent ( Figure 2C). Third, protein expression levels of mtDNA-encoded MTCO1 and nuclear DNA-encoded mitochondrial respiratory chain components NDUFB8, SDHB, UQCRC2, and ATP5A were measured by western blot analysis. Compared with those of the control group, the expressions of mitochondrial respiratory chain components, both mtDNA-encoded and genomic DNA-encoded, were reduced in MTERF3-KD cells ( Figure 2D). These results indicate that MTERF3 positively regulates the expression profile of mitochondrial respiratory complexes in SH-SY5Y cells. Interestingly, knockdown of MTERF3 does not seem to have a major impact on cell growth or cell cycle of SH-SY5Y cells ( Figure 2E).

In addition, we also performed MTERF3 overexpression (OE) experiments in SH-SY5Y cells and monitored the replication, transcription, and translation of mtDNA. The mtDNA level showed a 29% increase upon MTERF3 overexpression compared with the control group ( Figure 2F). Next, mRNA levels of *CYTB*, *ND4L*, *ND6*, and *MTCO1* were measured, which showed an opposite trend compared with those in MTERF3-KD cells: mRNA levels of *CYTB*, *ND4L*, *ND6*, and *MTCO1* were significantly decreased by approximately 92.3%, 94.5%, 90.7%, and 92.6% respectively ( Figure 2G), consistent with the negative regulator role of MTERF3 in mtDNA transcription. Finally, protein expression levels of MTCO1, NDUFB8, SDHB, UQCRC2, and ATP5A were measured by western blot analysis which showed the same decreasing trend although to a lesser extent compared with those in the MTERF3-KD group ( Figure 2H).

### MTERF3 plays a protective role in MPP+-induced mitochondrial dysfunction

**Figure 3:**
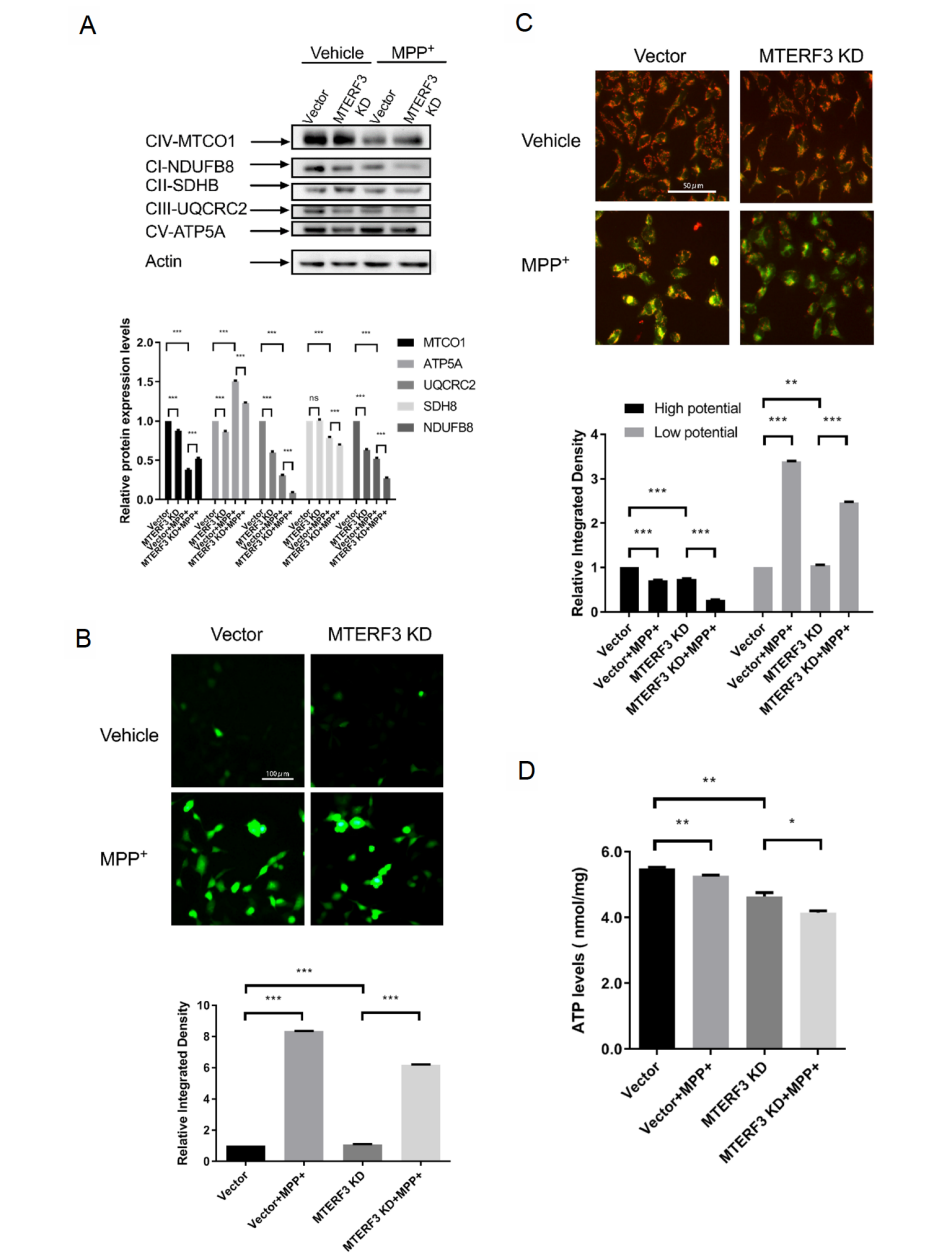
Figure 3 Effects of MTERF3 knockdown on MPP+-treated cells (A) Expressions of representative mtDNA and nuclear DNA-encoded proteins. (B) Measurement of reactive oxygen species, ROS level. (C) Measurement of mitochondrial membrane potential, MMP level. Red indicates high membrane potential and green indicates low membrane potential. (D) Measurement of cellular ATP levels. Data are presented as the mean±SEM, n=3. * P<0.05, ** P<0.01, *** P<0.001. ns: not significant.

To understand the role of MTERF3 in MPP+-induced mitochondrial dysfunction, knockdown experiments were performed using MTERF3 shRNA in MPP+-treated SH-SY5Y cells. After MPP+ treatment, expression of mtDNA-encoded MTCO1 showed a large decrease, which was slightly reversed by MTERF3 knockdown, opposite to the trend seen in the MPP+-untreated group. Expressions of the nuclear DNA-encoded NDUFB8, SDHB, UQCRC2, and ATP5A generally exhibited a decreasing trend in the MTERF3-KD group, in both the MPP+-untreated and MPP+-treated cases, which was more apparent in the latter case ( Figure 3A). The reduction of MTCO1 expression after MTERF3 knockdown in MPP+-untreated case might be related to the adverse effect of MTERF3 knockdown on ribosome biogenesis, affecting the expressions of both mtDNA and nuclear DNA-encoded mitochondrial proteins. Surprisingly, upon MPP+ treatment, MTERF3 knockdown seemed to boost the MTCO1 expression, suggesting that compensation effects might be activated by MPP+ treatment.

Next, the effect of MTERF3 knockdown on mitochondrial function was evaluated by monitoring the ROS level, mitochondrial membrane potential, and ATP formation. DCFH-DA probe experiments were used to detect the ROS level, which showed a significant increase after MPP+ treatment. There seemed to be a noticeable increase in ROS level in the MTERF3-KD group compared with that in the control group in the MPP+-untreated case, although not in the MPP+-treated case ( Figure 3B). JC-1 probe experiments were performed to detect the change in MMP level. The red fluorescence intensity of both groups of cells was significantly decreased and the green fluorescence intensity was significantly increased after MPP+ treatment, indicating that mitochondrial membrane depolarization occurred in both the control and MTERF3-KD groups. In the MPP+-treated case, the mitochondrial membrane depolarization seemed to be worsened in the MTERF3-KD group compared with that in the control group ( Figure 3C). The effect of MTERF3 knockdown on ATP formation was also measured using the ATP detection assay. It was found that ATP level in the MTERF3-KD group was decreased in both the MPP+-treated and MPP+-untreated cases ( Figure 3D), indicating mitochondrial malfunction in the absence of MTERF3. These results suggest that MTERF3 may play a generally protective role in MPP+-induced mitochondrial dysfunction.

**Figure 4:**
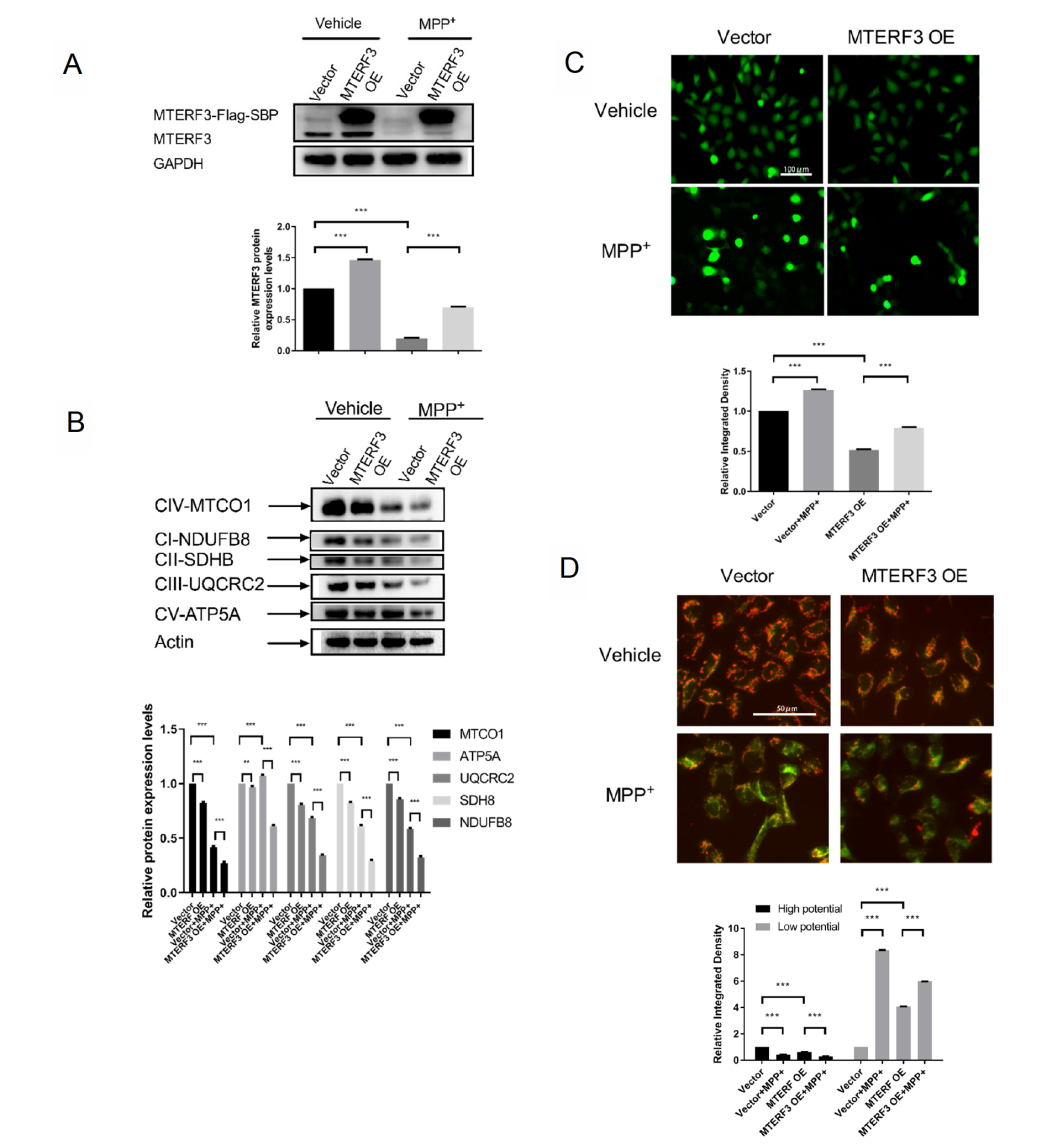
Figure 4 Effects of MTERF3 overexpression on MPP+-treated cells (A) MTERF3 protein expression. Bar plots show the expression of endogenous MTERF3. (B) Expressions of representative mtDNA and nuclear DNA-encoded proteins. (C) Measurement of ROS level. (D) Measurement of MMP level. Red indicates high membrane potential and green indicates low membrane potential. Data are presented as the mean±SEM, n=3. * P<0.05, ** P<0.01, *** P<0.001. ns: not significant.

In addition, MTERF3 overexpression experiments were also performed. As expected, a significant increase in expression of exogenous MTERF3 was observed in MTERF3-OE cells compared with the control group in both MPP+-treated and MPP+-untreated cases ( Figure 4A). Interestingly, the endogenous MTERF3 expression was also slightly increased after MTERF3 overexpression. Expressions of mtDNA-encoded and nuclear DNA-encoded mitochondrial proteins were also measured. Compared with the control group, the MTERF3-OE group showed noticeable protein level decreases in mtDNA-encoded MTCO1, consistent with the negative role of MTERF3 in mtDNA gene expression. Similar to those in the MTERF3-KD group, the nuclear DNA-encoded NDUFB8, SDHB, UQCRC2, and ATP5A levels in the MTERF3-OE group also exhibited a decrease in protein expression levels in both MPP+-treated and MPP+-untreated cases ( Figure 4B). In addition, upon MPP+ treatment, MTERF3 overexpression seemed to reduce MTCO1 expression similar to the MPP+-untreated case, suggesting that compensation effects were not activated by MPP+ treatment in the MTERF3-OE group.

The effect of MTERF3 overexpression on mitochondrial function was also evaluated in the MTERF3-OE group. DCFH-DA probe experiments showed a noticeable decrease in ROS level in the MTERF3-OE group compared with that in the control group in both the MPP+-untreated and MPP+-treated cases ( Figure 4C). For JC-1 experiments, the red fluorescence intensity of both groups of cells was significantly decreased and the green fluorescence intensity was significantly increased after MPP+ treatment, indicating that mitochondrial membrane depolarization occurred in the control group and in the MTERF3-OE group. Interestingly, in the MPP+-treated case, the mitochondrial membrane potential (high potential) seems to be less worsened in terms of the percentage decrease in the MTERF3-OE group (30%) compared with that in the MTERF3-KD group (63%) ( Figure 4D). Similar to the knockdown experiments, overexpression experiments also suggest that MTERF3 plays a generally protective role in MPP+-induced mitochondrial dysfunction.

## Discussion

One surprising finding in this study is that both MTERF3 knockdown and overexpression caused a decrease in mtDNA-encoded MTCO1 expression. The explanation for the seemingly contradictory results resides in the work of Wredenberg *et al*. [12] and Roberti *et al*. [25], which raised the interesting possibility that MTERF3 may act on both transcription and translation of mtDNA genes such as *MTCO1*. In the MTERF3-KD group, although the lack of MTERF3 boosts the *MTCO1* mRNA level, it also impairs the assembly of the large subunit of the mitochondrial ribosome and inhibits protein translation more significantly. The combined effect in the MTERF3-KD group is thus to decrease MTCO1 expression. In the MTERF3-OE group, the *MTCO1* mRNA level is decreased, causing decreased expression of MTCO1. But since MTERF3 is abundant, the assembly of the large subunit of the mitochondrial ribosome is in normal state with protein translation almost unaffected. The combined effect of MTERF3-OE group is thus also to decrease MTCO1 expression.

As a negative regulator of mtDNA replication [26] and transcription [11], it is not surprising to find that MTERF3 knockdown increases mtDNA replication. But the finding that MTERF3 overexpression also increases mtDNA replication is quite surprising. We argue that MTERF3 overexpression may trigger a negative feedback loop to maintain mitochondrial homeostasis similar to the negative feedback loop identified for mitochondrial transcription-regulating factor, TFAM [27].

It has been shown that mitochondrial homeostasis is perturbed in SH-SY5Y cells under MPP+ treatment in terms of changes in mtDNA replication and translation [11]. In this study, we tested whether the effect of MPP+ is correlated with MTERF3 expression. We found that MPP+ treatment significantly downregulated the MTERF3 expression in the cellular model, which is consistent with our previous findings in the MPP+-induced mouse model, where MPTP (metabolized to MPP+ *in vivo*) treatment downregulated the MTERF3 expression in mice brain significantly [24]. Both the animal model and cellular model suggest that MTERF3 may play a regulatory role in mitochondrial dysfunction.

Mitochondrial function impairment in SH-SY5Y cells was indicated by significantly elevated ROS levels and reduced MMP levels in this study, particularly after MPP+ treatment. The discrepancy of ROS or MMP levels between low and high MTERF3 expression can be quite small. This finding may be because of the possibility that mtDNA replication and translation are regulated not only by MTERF3 but also by other MTERF family members. It is possible that the expressions of other MTERF family members are also perturbed under MPP+ treatment, counteracting the effects of MTERF3. Therefore, while changes in MTERF3 expression can exacerbate the abnormality of translation of mtDNA induced by MPP+, they may not be sufficient to have a sizable impact on MPP+-induced mitochondrial dysfunction. Evidence supporting this hypothesis includes studies showing that while transcription factors NRF1 (nuclear respiratory factor 1) and NRF2 are mainly responsible for transcribing regulatory mitochondrial proteins such as MTERF family members, the promoter regions of MTERF family members can be quite different [ 28, 29] . For instance, while the promoter region of both *MTERF1* and *MTERF2* contains NRF-2 binding site, the promoter region of *MTERF3* does not contain any NRF-2 binding site. Therefore, even if the environmental toxin MPP+ can act on transcription factors such as NRF-2, MTERF family members are likely subject to a diverse array of transcriptional regulation pathways, which may produce either congruent or opposing effects on mitochondrial function.
